# Implications of updated protocol for classification of childhood malnutrition and service delivery in world’s largest refugee camp amid this COVID-19 pandemic

**DOI:** 10.1017/S1368980022000052

**Published:** 2022-01-12

**Authors:** Afsana Anwar, Probal Kumar Mondal, Uday Narayan Yadav, Abu Ahmed Shamim, Abu Ansar Md. Rizwan, Sabuj Kanti Mistry

**Affiliations:** 1 Health and Nutrition, Social Assistance & Rehabilitation for the Physically Vulnerable (SARPV), Cox’s Bazar, Bangladesh; 2 National Centre for Epidemiology and Population Health, Research School of Population Health, The Australian National University, Canberra, ACT, Australia; 3 Center for Research Policy and Implementation, Biratnagar, Nepal; 4 Centre for Primary Health Care and Equity, University of New South Wales, Sydney, NSW 2052, Australia; 5 BRAC James P Grant School of Public Health, BRAC Univesity, Dhaka 1213, Bangladesh; 6 ARCED Foundation, 13/1, Pallabi, Mirpur-12, Dhaka, Bangladesh; 7 Department of Public Health, Daffodil International University, Dhaka 1207, Bangladesh

**Keywords:** Rohingya, Weight-for-height z-score, Mid upper arm circumference, Moderate acute malnutrition, Severe acute malnutrition

## Abstract

**Objectives:**

During the COVID-19 pandemic, the authorities made a change in the classification of malnutrition and concomitant service delivery protocol among the Rohingya children, residing in world’s largest refugee camp, located in Cox’s Bazar, Bangladesh. In this paper, we discussed the potential implications of this updated protocol on the malnutrition status among children residing in the Rohingya camps.

**Design:**

This paper reviewed relevant literature and authors’ own experience to provide a perspective of the updated protocol for the classification of malnutrition among the children in the Rohingya camps and its implication from a broader perspective.

**Setting:**

Rohingya refugee camps, Bangladesh.

**Participants:**

Children aged less than five years residing in the Rohingya camps.

**Results:**

Major adaptation during this COVID-19 was the discontinuation of using weight-for-height z-score (WHZ) and the use of only mid upper arm circumference (MUAC) and presence of oedema for admission, follow-up and discharge of malnourished children in the camps. However, evidence suggests that use of MUAC only can underestimate the prevalence of malnutrition among the children in Rohingya camps. These apparently non-malnourished children are devoid of the rations that they would otherwise receive if classified as malnourished, making them susceptible to more severe malnutrition.

**Conclusions:**

Our analysis suggests that policymakers should consider using the original protocol of using both MUAC and WHZ to classify malnutrition and retain the guided ration size. We also believe that it would not take an extra effort to adopt the original guideline as even with MUAC only guideline, certain health measures needed to adopt during this pandemic.

COVID-19 pandemic is the greatest public health calamity that the world facing today, which is causing thousands of deaths every day around the globe^(1)^. Although everyone is at risk of the detrimental effect of COVID-19, it can be a serious concern for children and young people as it may hamper their future growth and development^(2)^. This is truly expressed by UNICEF Executive Director Henrietta Fore as, *‘Don’t let the children be the hidden victims of COVID-19 pandemic’* ^(3)^.

Displaced and refugee populations are particularly at risk of this jeopardy because of their residence in densely populated refugee camps with social distancing often impossible to follow^(4)^ as well as limited health, sanitation and hygiene facilities available at camps^(5)^. The Rohingya people, also referred to as Forcibly Displaced Myanmar Nationals, are Muslim minorities of Myanmar who flee in large numbers from their land since August 2017^(6)^ and are now settled in Cox’s Bazar, Bangladesh, in the largest refugee camp in the world^(7)^. Like other refugee camps of the world, Rohingya camps are also overpopulated and have limited access to health facilities, making the people residing in these camps vulnerable to coronavirus infection^(8)^.

According to UNHCR Operational Data Portal, the total population in Rohingya camps is 889 704, of which nearly one-fifth (18·7 %) are young children aged less than five years^(9)^. The high rate of malnutrition has been a great concern among the young children in Rohingya camps. The most recent Standardized Monitoring and Assessment of Relief and Transitions survey carried out in October 2017 recorded a high (19 %) malnutrition rate as classified by weight-for-height z-score (WHZ score), where the prevalence of severe acute malnutrition and moderate acute malnutrition (MAM) was 3 % and 16 %, respectively^(10)^.

In the meantime, considering the social distancing rules imposed by the camp authority due to COVID-19 pandemic, Refugee Relief and Repatriation Commissioner and agreement among the technical advisors of three UN agencies (UNHCR, UNICEF and WFP)^(11)^, the nutrition sector redefined the classification of malnutrition of the children and related safety measures and service delivery protocols^(11,12)^. In this paper, we discussed the implications of this new protocol on overall malnutrition status among the young children in Rohingya camps amid this COVID-19 pandemic.

## Adaptation in nutrition service delivery during the COVID-19 pandemic situation

Nutrition sector is coordinating and administrating emergency nutrition response since the beginning of the humanitarian crisis in Cox’s Bazar. At present, nutrition service is being provided from forty-six integrated nutrition facilities in thirty-four camps. One-stop-shop approach was taken to ensure access of all nutrition-related services from one place, including outpatient therapeutic programme, therapeutic supplementary feeding programme and blanket supplementary feeding programme.^(11)^ Before the COVID-19 pandemic, admission and discharge criteria and other services were conducted according to the National Guidelines for Community Based Management of Acute Malnutrition. Ready to use therapeutic food was provided based on weight during every weekly visit in outpatient therapeutic programs, while children are provided with one sachet of ready to use supplementary food in each 14 d according to the therapeutic supplementary feeding programme protocol^(13)^. The detail admission and discharge criteria are presented in Table 1.

**Table 1:** Admission and discharge criteria for OTP and TSFP^(13)^

Situation	Admission criteria for OTP	Discharge criteria for OTP
6–59-months aged SAM children	Bilateral oedema: +/++ or	No bilateral oedema or resolving oedema
	WHZ < –3 Z score and /or MUAC < 11·5 cm and presence of appetite and absence of medical complications as per National CMAM/IMCI Protocol	For those admitted based on WHZ > –3 Z score and/or for those admitted based on MUAC, MUAC ≥ 11·5 cm and presence of appetite and absence of medical complications as per National CMAM/IMCI Protocol for consecutive visits since admission
6–59-month aged MAM children	WHZ < –2SD to ≥ –3SD and/or MUAC: 11·5 cm –12·4 cm and without medical complications, as per national Integrated Management of Childhood Illness (IMCI) protocol and absence bilateral oedema.	For those admitted based on WHZ > –2 Z score for 2 consecutive visits or for those admitted based on MUAC, MUAC greater than or equal to 12·5 cm for consecutive visits since admission

CMAM, community-based management of acute malnutrition; IMCI, integrated management of childhood illness; MAM, moderate acute malnutrition; SAM, severe acute malnutrition; MUAC, mid upper arm circumference; WHZ, weight for height z-score.

As part of the COVID-19 response management, the protocol for nutrition programs delivered in the camps was reviewed to identify potential source of coronavirus infection resulted through program implementation. In line with this, UNICEF Global Nutrition Cluster and Global Technical Assistance Mechanism for Nutrition proposed to avoid using weight-for-height z-scores (WHZ) for diagnosis of severe malnutrition on 27 March 2020 because it has the potential to spread contamination during height and weight measurement. Concomitantly, with the guidance of technical advisors of all three UN institutions at headquarters, regional and national levels, and in line with international recommendations, the Nutrition Sector agreed to use only mid upper arm circumference (MUAC) and oedema for testing, admission, tracking and discharge of children aged 6–59 months in all Community Based Management of Acute Malnutrition programs within the camps. Moreover, local decision was made by Nutrition Sector partners to raise the standard of MUAC, influenced by the results of the Cox’s Bazar Standardized Monitoring and Assessment of Relief and Transitions general survey, which showed poor agreement between MUAC and WHZ, and other children with MUAC up to 135 mm were identified as malnourished using WHZ. To ensure that no children were missed, the final MUAC cut-off was agreed to be < 120 mm for outpatient therapeutic program and ≥ 120 mm and < 130 mm for targeted child feeding programs. Therefore, the major adaptation during this COVID-19 was the discontinuation of using weight scales and height board and use of only MUAC and presence of oedema for admission, follow up and discharge in all Community Based Management of Acute Malnutrition programming in both makeshift and registered camps^(11,12)^ (Table 2). The ration protocol was changed for outpatient therapeutic programme to biweekly from weekly and biweekly to monthly for therapeutic supplementary feeding programme. The ration protocol was also changed to 2 Ready to use therapeutic food per day for 6–23-month aged children and 3 Ready to use therapeutic food per day for 6–59-month aged children^(12)^. Moreover, capacity-building initiative of mothers and caregivers known as ‘Mother-led MUAC’ was undertaken so that caregivers can measure their children independently^(12)^.

**Table 2:** Updated admission criteria during COVID-19^(12)^

		OTP	TSFP
New admissions	Admission criteria for new cases	SAM without medical complications children < 12·0 cm and/or grade + or grade ++ oedema	MAM child children MUAC 12·0 cm–< 13·0 cm

OTP, outpatient therapeutic programme; TSIP, therapeutic supplementary feeding programme; SAM, severe acute malnutrition; MAM, moderate acute malnutrition; MUAC, mid upper arm circumference.

## Implications of changes in broader perspective

The concordance between WHZ and MUAC for assessing malnutrition depends on several factors and varies between countries and regions, and the use of one criterion can both underestimate and overestimate the prevalence of malnutrition. It is recommended for the countries of Southern Asia, and The SAHEL region (spread between Sahara Desert to the north and tropical savannas to the south) to maintain WHZ criteria for assessing malnutrition^(14)^. A recent study^(15)^ carried out in twelve districts of Bangladesh also shows the dis-concordance between MUAC and WHZ and suggests increasing cut-off of the MUAC criteria to increase the chance of covering the malnourishment classified by WHZ. However, it comes with the inclusion of non-wasted children and MAM children in severe acute malnutrition treatment program^(15)^. According to the Standardized Monitoring and Assessment of Relief and Transitions survey final report of round 4, the Global Acute Malnutrition (GAM) rate by WHZ was 10·9 %, 13·3 % and 12·1 %, respectively, which was only 4·9 %, 2·7 % and 2·2 % respectively by MUAC only, and the combined GAM rate by WHZ and MUAC was 12·2 %, 13·6 % and 12·1 %, respectively, in Rohingya camps^(10)^. This suggests that the prevalence of GAM by WHZ and combined WHZ and MUAC concordance far better than MUAC only. Other studies carried out in similar setting also support this finding^(16,17)^. Therefore, it is obvious that many of the children in camps who are not being classified as malnourished according to the MUAC only criteria would have been categorised as malnourished if they are assessed according to the recommended WHZ or combined WHZ and MUAC criteria. It is a great concern that these apparently non-malnourished children are devoid of the rations that they would otherwise receive if classified as malnourished, making them susceptible to more severe malnutrition.

Moreover, due to the changed ration protocol, the frequency of visiting integrated facilities has been reduced. For example, previously, the severe acute malnutrition children were visited in the facility four times in a month on 7 d interval and MAM children twice in a month on 14 d interval. Due to changed protocol, now the severe acute malnutrition children are visited twice in a month on 14 d interval and MAM children once in a month on 28 d interval. Authors’ own experience from the field suggests that this has led to an increased length of stay that might result in delayed rehabilitation of the children. Furthermore, this low frequency of visits at the facility has limited the monitoring at field level, which might have an effect on programme performance. However, average weight gain and growth monitoring promotion are also being suspended due to MUAC only plus oedema criteria. But both are important indicators for monitoring growth and nutritional status, particularly among older children aged 24–59 months, among whom change of MUAC is less prominent than change of weight compared with those of younger children aged 6–23 months.

## Way forward

Based on the earlier discussion, we believe that it is important that the policymakers immediately consider using the original protocol of using both MUAC and WHZ and ration size after ensuring coronavirus infection control and other necessary arrangements for services delivery amid this pandemic. This would help reduce the risk of underestimating the actual prevalence of malnutrition that may result using MUAC only criteria. It is also important to note that even for the measurement of MUAC, certain health measures need to be taken during this COVID-19 pandemic and therefore, we believe, it is possible to go back to the original guideline with limited extra effort. From authors’ own direct field observation and experience, it was also obvious that replacing the original protocol in the real-world implementation will not incur an extra cost, time, and resources as the institutional arrangements to implement the original protocol is already in place in the field. We also found instances where both WHZ and MUAC have been used in refugee camps amid this pandemic. For example, in the refugee camps of Sudan, the authorities continued to use both measures (MUAC and Z scores and bilateral pitting oedema) for admission of Community Based Management of Acute Malnutrition in case of without any restrictions in population mobility (night curfew)^(18)^. Likewise, at the therapeutic feeding centre of the refugee settlements of Yemen, where all the infection, prevention and control material and PPE are available for safe nutritional assessment, WHZ and oedema are assessed, and treatment is provided according to the national protocol^(19)^. In refugee camps of Uganda, extended MUAC thresholds of 12·0 cm to 13·0 cm are considered ‘at risk’ for admission, and after admission, weight is taken to calculate ration size^(20)^.

## Conclusion

It is conspicuous from the above discussion that MUAC-only protocol adopted for classifying malnutrition during this pandemic can underestimate the prevalence of malnutrition among young children in Rohingya refugee camps, along with other service-level changes that occurred during this pandemic further exacerbate the malnutrition condition of the children in camps. We propose that policymakers and public health practitioners immediately consider adopting the previous guidelines of classifying malnutrition and revise the service delivery modality by considering COVID-19 issue to make it more streamlined and prevent large-scale malnutrition among children in camps. It is also recommended to conduct a study to determine an appropriate cut-off point for child MUAC in the Rohingya camps.
